# Genome-wide identification and expression analysis of the mating-responsive genes in the male accessory glands of *Spodoptera litura* (Lepidoptera: Noctuidae)

**DOI:** 10.1186/s43141-023-00466-0

**Published:** 2023-02-01

**Authors:** R. Mamtha, Tannavi Kiran, Vivek Chandramohan, B. S. Gowrishankar, D. Manjulakumari

**Affiliations:** 1 Department of Microbiology & Biotechnology, Bangalore University, Bengaluru, Karnataka 560056 India; 2 Department of Biotechnology, Siddaganga Institute of Technology, Tumakuru, Karnataka 572103 India

**Keywords:** *Spodoptera litura*, Male accessory glands, RNA Seq, Mating-responsive genes

## Abstract

**Background:**

Mating elicits significant changes in gene expression and leads to subsequent physiological and behavioural modifications in insects. The reproductive success of both sexes is contributed immensely by the male accessory gland (MAG) proteins that are transferred along with sperms to the female reproductive tract during mating where they facilitate several processes that modify the post-mating behaviour. The mating-responsive genes in the MAGs have been identified and reported in many insects but have not been well-characterized in the important agricultural pest *Spodoptera litura*. Here, we present RNA sequencing analysis to identify mating-responsive genes from the accessory glands of virgin males and males interrupted during mating.

**Results:**

Overall, 91,744 unigenes were generated after clustering the assembled transcript sequences of both samples, while the total number of transcripts annotated was 48,708 based on sequence homology against the non-redundant (NR) database. Comparative transcriptomics analysis revealed 16,969 genes that were differentially expressed between the two groups, including 9814 up-regulated and 7155 down-regulated genes. Among the top 80 genes that were selected for heat map analysis, several prominent genes including odorant binding protein, cytochrome P450, heat shock proteins, juvenile hormone binding protein, carboxypeptidases and serine protease were differentially expressed.

**Conclusions:**

The identified genes are known or predicted to promote several processes that modify the female post-mating behaviour. Future studies with the individual MAG protein or in combination will be required to recognize the precise mechanisms by which these proteins alter female physiology and reproductive behaviour. Thus, our study provides essential data to address fundamental questions about reproduction within and among insects and also paves way for further exploration of the functions of these proteins in female insects.

## Background

In many insects, mating initiates a behavioural and physiological change in females, further triggering responses in numerous processes related to fertility. These changes in the female are the consequences of receipt of MAG secretion, a mixture of protein and other products. During mating, the MAG secretions along with sperms are transferred to the female reproductive tract where they facilitate processes such as sperm protection, competition, storage and activation [24, 39], at the same time they modify female post-mating behaviour by decreasing female receptivity to remate, increasing egg production and also affecting longevity in females [3, 61]. Additional functions of MAG secretions that indirectly affect the female reproductive behaviour and physiology include modulation of host-seeking and feeding behaviour, production of antibacterial proteins and alteration of metabolism.

In recent years, with the advent of transcriptomics and proteomic methods, comprehensive studies aimed at the identification and analysis of MAG proteins and the encoding genes in insects belonging to different orders, such as *Aedes aegypti* [12], *Drosophila melanogaster* [50], *Apis mellifera* [4], *Dermacentor variabilis* [51], *Tribolium castaneum* [52], *Teleogryllus oceanicus* [6], *Bactrocera dorsalis* [55], *Callosobruchus maculatus* [8], *Crematogaster osakensis* [23] and *Anastrepha ludens* [49]. Most of the post-mated changes induced by MAGs in females have been reported to trigger substantial expression of genes involved in metabolic processes, catalytic activity, nucleic acid binding and immune response. Besides those, the genes associated with mating also comprise proteases, heat shock proteins, putative chemosensory proteins, detoxification enzymes and housekeeping genes. It will be interesting to identify such mating-responsive genes and their regulatory mechanisms in *Spodoptera litura*, a polyphagous pest insect.

In the Asian tropics, the tobacco cutworm *Spodoptera litura* (Lepidoptera: Noctuidae) is a widespread pest attacking more than 120 crops belonging to 44 families [43], of which 40 species are known from India alone [21]. *S. litura* has also been reported to be resistant to all classes of insecticides viz., organochlorines, organophosphate and synthetic pyrethroid [1, 29, 56]. Being a nocturnal insect, all the reproductive-related activities take place during the night, wherein maximum mating occurs during the second night of emergence [31]. Reproductive success in the common cutworm is attainable by a most efficient reproductive system, while its efficiency contrarily results from the synchronization of the endocrine system that controls reproduction with both environmental signals and the internal physiological state of the insect. However, the functional characterization of mating-responsive genes in *S. litura* remains poorly understood so far, thus limiting our knowledge of its reproductive biology.

Our previous studies have shown that MAG proteins induce significant changes in female reproductive behaviour. At first, comparative studies of virgin and mated MAG proteins on SDS-PAGE analysis revealed the presence and absence of a protein <14 kDa in virgin and mated moths respectively, indicating the transfer of protein to female moths during mating [32]. Further on, using 2D gel electrophoresis and mass spectrometry, several potential molecules that modulate the female reproductive physiology and behaviour were characterized [33]. Subsequently, a recombinant allatotropin was revealed to induce egg-laying in virgin female moths of *S. litura* [34]. The genome of *S. litura* has also been sequenced and published by Cheng et al. [14], thus providing resources for the identification of several mating-responsive genes. Based on the previous investigations in *S. litura* and other insects, the present study was conceived to exemplify the mating-responsive genes from the accessory glands of virgin males and males interrupted during mating through RNA-Seq technology.

## Methods

### Insect rearing

The parental stock of *Spodoptera litura* (National Accession No. NBAII-MP-NOC-02) was obtained from the National Bureau of Agricultural Insect Resources, (NBAIR, erstwhile PDBC, ICAR), Bengaluru. The insects were reared on an artificial diet [16] under controlled conditions in the laboratory at 25± 2°C with 45±5% relative humidity and 12:12 (L:D) photoperiods. The pupae collected were sexed and maintained separately according to their distinct morphological characters. Cotton dipped in 10% honey solution was provided as food to the adult moths soon after emergence.

### Tissue collection and RNA isolation

For transcriptome sequencing, accessory glands were collected from 0-day-old to 14-day-old virgin males after 2 h of the onset of scotophase and later pooled as a single sample for RNA extraction (control). The tissues from males, interrupted at 30 min during mating, were collected (experimental), as mating lasts for 40 min. These samples were immediately flash-frozen in liquid nitrogen. Total RNA was isolated by following the guanidine isothiocyanate method as described by Ribaudo et al. [46] with slight modifications, carried out in ribonuclease (RNase)-free environment. Agilent Tape station D1000 was used for determining the RNA integrity number (RIN) and a Qubit fluorometer was used for RNA quantification.

### Generation of cDNA library and sequencing

RNA sample with a RIN value above seven was used for cDNA synthesis. Equal amounts of high-quality RNA from accessory gland tissues of virgin males and males interrupted during mating were taken for cDNA synthesis. The cDNA library construction was done using NEB Next Ultra RNA Library Prep Kit and Illumina sequencing of the samples was performed at Sandor Life Sciences Pvt. Ltd. (Hyderabad, India). The mRNA was purified from 1 μg of the total RNA using Oligo dT beads and was fragmented into short sequences at the appropriate temperature. These fragments served as templates and the first strand of cDNA was synthesized using random primers and reverse transcriptase, followed by the synthesis of the second-strand cDNA using RNaseH and DNA polymerase I. After end-repair, the fragments were ligated using sequencing adaptors and purified using AMPure XP beads. These adaptor-ligated fragments were amplified using PCR and the products were further purified using AMPure XP beads to create a cDNA library and were assessed on the Agilent Bioanalyzer 2100 system. Quantification and size distribution of the prepared library was determined using a Qubit flourometer and Agilent Tape station D1000 Kit according to the manufacturer’s instructions. The resulting cDNA libraries were then paired-end sequenced (2 × 150 bp) with the Illumina HiSeq^TM^ platform.

### De novo assembly and gene annotation

Clean short reads were obtained from all the 150-bp paired-end raw reads by removing the adapter sequences and low-quality bases. Quality check and processing of the short reads were done using in-house Perl scripts. The processed reads with a minimum contig length of 300 bases were assembled into transcripts for the samples independently using Trinity software (Trinityrnaseq-r20140413p1). To generate full-length transcripts, homologous contigs with 95% identity were clustered and aligned using CD-HIT EST (v4.6.1) tool and Bowtie2-2.2.4 respectively. The resulting sequences deemed as unigenes generated after clustering the merged sequences from the two samples were aligned to NCBI non-redundant protein database and a BLAST search was conducted. All sequences with significant blast hits (e≤10^−3^) were then mapped and annotated using the Diamond tool, in which the proteins with the highest sequence similarities along with their functional annotations were retrieved. Sequentially after Gene Ontology (GO) annotation, the GO functional classification was obtained using the panther classification system and KAAS (KEGG Automatic Annotation Server) was used to identify the biological pathways involved using *Bombyx mori*, also a lepidopteran, as reference organism [37]. Furthermore, sequences containing a secretion motif were identified using SignalP 4.0 [40].

### Identification of differentially expressed genes

To determine the specific expression of genes, the reads for the samples (control and experimental) were separately aligned to the unigene sequences and fragments per kb per million fragments (FPKM) values were used to determine expression levels. The read count profiles for each gene were tested using DESeq “R” package. The package DESeq provides methods to test for differential expression by use of the negative binomial distribution and a shrinkage estimator for the distribution’s variance. For all comparisons, genes were considered significantly differentially expressed if the expression log2 fold change of up-regulated and down-regulated genes were ≥2 and ≤-2 respectively. Default parameter settings (*p*-value cut-off for false discovery rate 0.001) in the DESeq package were then used for the final differentially expressed gene (DEG) analysis to generate outputs in the form of a heat map.

## Results

### Sequence assembly

Two cDNA libraries derived from the MAGs of *S. litura* virgin males (control) and males interrupted during mating (experimental) were constructed. The transcriptome sequencing provided approximately 46,381,012 and 71,105,012 paired-end reads for the control and experimental sample respectively. After removing reads containing adapter sequences, poly-N reads and low-quality reads from the raw data, approximately 45,743,830 and 70,146,494 clean reads were obtained from each sample (Table 1). All high-quality reads from the two samples were pooled and assembled using Trinity. A total of 99,633 transcripts with lengths longer than 300 bp were generated.

**Table 1:** Summary of *S. litura* transcriptome assembly

Transcriptome assembly	Virgin males	Males interrupted during mating
Transcripts generated	53,029	46,604
Maximum transcript length	31,225	23,622
Minimum transcript length	300	300
Average transcript length	838.211	746.455
Total transcript length	44,449,486	34,787,811
Total number of non-ATGC characters	0	0
Percentage of non-ATGC characters	0	0
Transcripts > 100 b	53,029	46,604
Transcripts > 500 b	26,218	22,048
Transcripts > 1 Kb	12,139	9227
Transcripts > 10 Kb	19	15
Transcripts > 100 Kb	0	0
Number of reads used	19,573,785	30,720,657
Total number of reads	22,871,915	35,073,247
Percentage of reads used	85.58%	87.59%

### Functional annotation and classification of the clustered transcripts

After clustering, the assembled transcript sequences of both the samples 91,744 unigenes were generated, out of which the total number of transcripts annotated was 48,708 based on sequence homology against the NR database. The highest number of unigene sequences matched with *Bombyx mori* (3855), followed by *Helicoverpa armigera* (341), and *Spodoptera litura* (265).

**Fig. 1:**
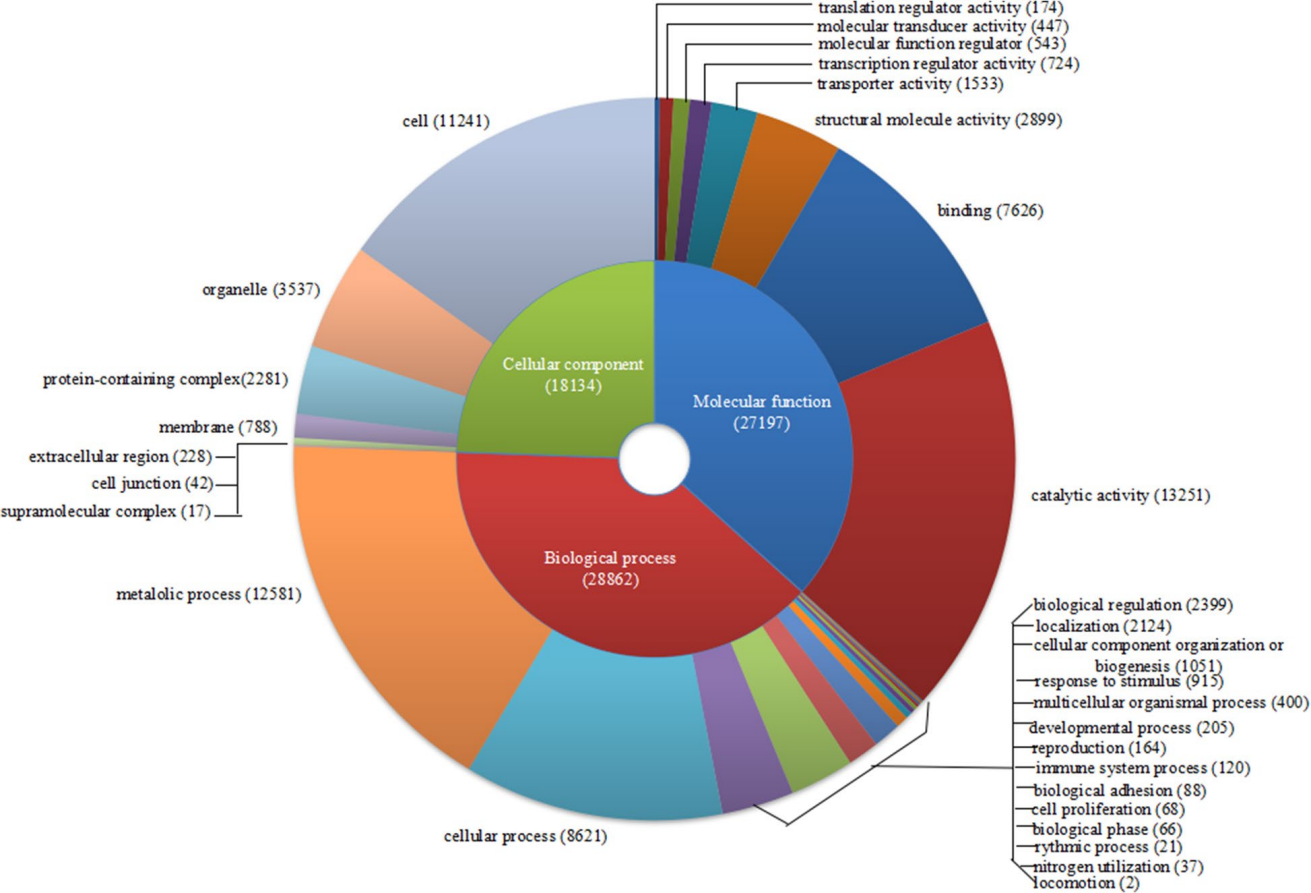
Gene ontology category distribution of MAG proteins in *Spodoptera litura*

Based on the Gene Ontology (GO) analysis, the unigenes were categorized into 31 functional groups of three ontological categories: biological process (28,862), cellular component (18,134) and molecular function (27,197). There were 12 functional groups that contained more than 1000 unigenes, including 16 biological processes, 7 cellular components and 8 molecular functions. The most dominant groups among the three ontological categories were metabolic process (12,581) and cellular process (8621) in biological process, catalytic activity (13,251) and binding (7626) in molecular function and cell (11241) in cellular component (Fig. 1). Then, all the unigenes were mapped to 394 predicted KEGG pathways, among which 57 pathways contained over 50 unigenes. The most abundant pathway was the metabolic pathway (675), followed by pathways of neurodegeneration-multiple diseases (224), and biosynthesis of secondary metabolites (222). On analysis by SignalP 4.0, we identified 2212 unigenes that contain signal sequences and are probably secretory. A major portion of these genes could not be identified and remained unannotated using the gene ontology tool, implying these proteins to be novel.

### Differential gene expression analysis and annotation

**Fig. 2:**
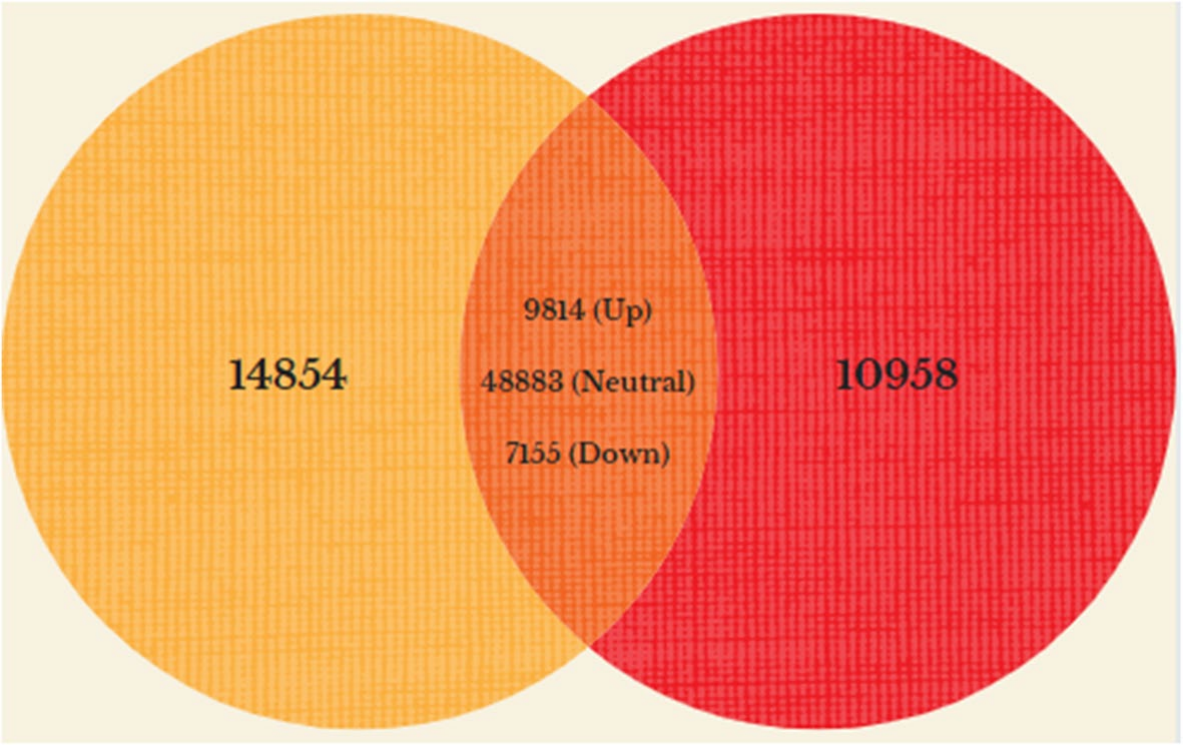
Venn diagram showing the number of up-regulated, down-regulated, neutral and unique transcripts

**Fig. 3:**
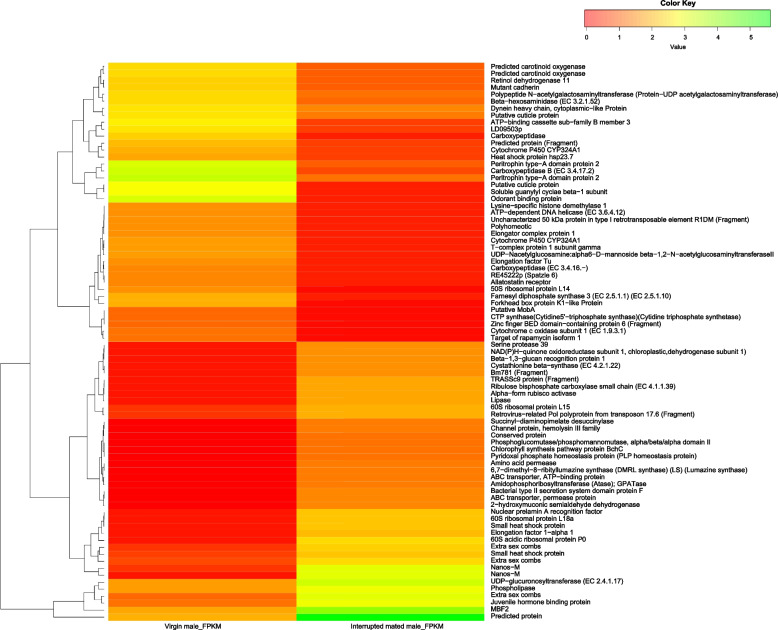
Heat map of top 80 differentially expressed transcripts across virgin males and males interrupted during mating. Red colour indicates down-regulated expression and green indicates up-regulated expression

To identify differentially expressed genes in the MAGs of virgin and mating-interrupted males, the transcript levels of both tissues were compared. In total, 16,969 unigenes were differentially expressed between the two groups, among which 9814 were up-regulated and 7155 down-regulated. In addition to the differentially expressed genes, 14,854 and 10,958 genes were unique to virgin and interrupted mated MAGs respectively (Fig. 2). For generating a heat map, the top 80 up- and down-regulated annotated transcripts were considered (Fig. 3).

## Discussion

Male accessory glands play a vital role in the reproduction of several insects. The secretions from MAGs transferred along with sperms to the female reproductive tract during mating facilitate several processes that modify female post-mating behaviour. Across diverse taxa, transcriptome and proteome analysis [5, 8, 20, 44, 51, 55] suggests that MAG proteins are transferred during mating affecting the reproductive success of both sexes and providing valuable information for a comprehensive understanding of insect reproduction. Thus, transcriptome analysis is one of the possible ways to identify the mating-responsive genes in the MAGs of *S. litura*.

Although the influence of proteinaceous components of MAGs on female reproductive behaviour has been widely demonstrated in several insects including *S. litura*, hitherto, the information about mating-responsive genes in this insect is limited. Here, the RNA isolated from the accessory glands of both virgin males and males interrupted during mating were sequenced to identify the unique genes as well as the differentially expressed mating-responsive genes in *S. litura*. Sequentially to confine the proteins expressed at any stage of the moth’s lifetime, accessory glands were collected from 0-day-old to 14-day-old virgin males after 2 h of the onset of scotophase and later pooled as a single sample for RNA extraction. Similarly, the tissues from males interrupted at 30 min during mating were collected, as mating lasts for 40 min [31], to identify transcriptome changes that were differentially expressed in moths interrupted during mating.

In this study, a high-quality transcriptome was generated from the MAGs of *S. litura* by next-generation sequencing technology. Overall, 91,744 unigenes were generated, of which 53.09% of transcripts were annotated based on the sequence homology against the NR database. The GO function categories of these unigenes were associated with cellular components, biological processes and molecular functions. Twelve functional groups contained more than 1000 unigenes, including 16 biological processes, 7 cellular components and 8 molecular functions. Enrichment of unigenes of KEGG pathways showed that the metabolic pathway was the significant enrichment, where most of these genes were involved in carbohydrate, energy and amino acid metabolism. Mating regulates several metabolism-related families of genes such as hydrolases, transferases and oxidoreductases. These genes from the MAGs are essential in the maintenance of stored sperm, egg production, immunity and detoxification [9, 42, 44, 53]. Besides the identification of the metabolism-related families of genes, 2212 unigenes containing signal sequences possibly secretory in nature were identified in this study. The majority of them remain unannotated when analysed using the gene ontology tool suggesting that the proteins could be novel.

DEG analysis generated from comparative transcriptomics is a proven tool to explore the mating-responsive genes in insects. In *S. litura*, among the 16,969 unigenes that were differentially expressed, 9814 up-regulated and 7155 down-regulated DEGs were identified in comparisons of virgin and interrupted mated MAGs respectively, while 10,958 genes were unique and expressed only during mating, providing valuable information on their role in reproduction. Our heat map analysis provides insights into the whole picture of gene expression of MAGs in virgins and males interrupted during mating. In this study, among the top 80 genes that were selected for heat map analysis, several prominent genes including odorant binding protein, cytochrome P450, heat shock proteins, juvenile hormone binding protein, carboxypeptidases and serine protease were differentially expressed. These genes are known or predicted to promote several processes that modify the female post-mating behaviour. In addition to those, the genes associated with reproduction also comprise transferases, hydrolases, lyases and oxidoreductases. Here, the possible functions of a few well-known genes that were differentially expressed during mating in the MAGs of *S. litura* have been discussed.

In several insect species, chemosensory-related genes in the females are known to be regulated by mating. Amongst them, odorant binding proteins (OBPs) are known to transfer the odorants and pheromones to their receptors assisting insects to choose several stimuli such as food sources, mates and oviposition sites [54, 62]. Odorant-binding proteins are mainly associated with olfaction; however, they are also identified in the male ejaculate of many insects [13, 26, 52]. For example, in *Bactocera dorsalis*, Wei et al. [58] detected several OBPs in the MAG secretions suggesting the transfer of OBPs to the female reproductive tract where they regulate the reproductive physiology in the females by binding to specific receptors. In *Aedes aegypti*, down-regulation of OBPs capable of switching the female host-seeking behaviour was reported [2]. Interestingly, in another study, knockdown of OBP22 in *A. aegypti* suggests that mating regulation of the gene could reduce the tendency for blood meal probing thereby decreasing the blood-feeding ability in females after mating [27, 48]. Furthermore, In *D. melanogaster* and *A. aegypti*, OBPs expressed in the MAGs were transferred to females during mating as potential modulators of female post-mating behaviour [19, 50]. Likewise, in this study, OBPs were found to be down-regulated in the MAGs of *S. litura* during mating suggesting they may be transferred to females during mating, aiding in similar roles as in other insects. Thus, OBPs may perhaps offer valuable insights for managing feeding and reproductive behaviour in insects.

Insect cytochrome P450s (CYPs) are mostly known to involve in the detoxification of xenobiotics and chemicals, including insecticides and plant allelochemicals [47]. It is plausible that detoxification of seminal fluid molecules by CYPs could impact the survival of female insects [36, 38, 59]. Moreover, the identification of CYPs in the MAGs revealed its function as a sperm storage protein in *D. melanogaster* [42] and as an antioxidant in *Anopheles gambaie* [17]. In several insect species, CYPs are recognized to control Juvenile Hormone III titers during Juvenile hormone biosynthesis crucial for reproductive maturity and mating behaviour [35, 57]. Similarly, the down-regulation of CPYs in MAGs of *S. litura* during mating could reflect several processes as indicated in other insects.

Heat shock proteins (Hsps) are generally synthesized in response to stress and also impact the susceptibility of insects to pathogens. These proteins are generally regulated by mating in several insect species including *D. melanogaster*, *Bemisia tabaci* and *A. mellifera* [25, 28, 36]. Hsps have also been identified in the MAGs of *A. gambaie* [7, 17], *A. mellifera* [5, 22] and *Aedes albopictus* [10]. Hsp70 and Hsp90 identified in the MAGs of *H. armigera* play a vital role in immune response while the suppression of Hsps during feeding in *A. aegypti* [9] leads to fewer eggs produced eventually. Three Hsps identified in MAGs of *S. litura* may play a similar role as in other insects, where HSP 23.7 is known to be down-regulated and two small heat shock proteins were reported to be up-regulated during mating.

Juvenile hormone (JH), an important regulator of insect morphogenesis and reproduction, is transferred from the synthesis site to the target tissues by a hemolymph carrier called juvenile hormone-binding protein (JHBP). JH molecules are mostly protected from hydrolysis by JHBP through the action of non-specific esterases. The transfer of JH from the MAGs to the female ovaries during mating is well demonstrated in *A. aegypti* [11]. A study in *B. dorsalis* revealed JHBP (CG5867) as one of the most abundant and highly expressed proteins in the MAGs that may regulate reproductive physiology when transferred to females [58]. In our study, up-regulation of JHBP signifies the role of the protein in transporting JH from the MAGs to the female reproductive tract during mating, as JH titers are crucial for reproductive maturity and post-mating behaviour in several insects.

Serine proteases and carboxypeptidases are common accessory gland proteins known to play a role in various aspects of insect reproduction. In many organisms, proteases and peptidases have been implicated in increased egg-laying after mating and are also linked to sperm activation and immune response. These genes are thought to protect females against male-derived pathogens [8, 30, 45, 60]. In *D. melanogaster*, proteases induced by MAGs are required for activation of the ovulation process in the female flies [30], whereas in the mosquito *A. aegypti*, carboxypeptidases are required for oocyte development [15]. The most significant example is the reported increased expression of a serine protease in *Drosophila* females that triggers the release of the mature sex peptide transferred by the male to the female during mating [41]. In *Dermacentor variabilis*, 12 different serine proteases and carboxypeptidases identified in the MAG transcriptome were known to play important roles in post-mating activities [51]. In *Lacanobia oleracea*, the depletion of Angiotensin I-converting enzyme (ACE), dipeptidyl carboxypeptidases in mated males and concomitant increase in ACE activity in the bursa copulatrix and spermatheca of the female suggested the transfer of ACE from the male to the female during mating [18]. The down-regulation of serine protease and carboxypeptidases in the MAGs of *S. litura* suggests that these proteins could be passed to the females during mating thereby inducing post-mated changes in female moths.

Identification of all the key genes discussed above may play different roles in post-mated females to ensure successful fertilization. In a previous study on *S. litura*, we revealed the down-regulation of protein/s in MAGs of mated moths and moths interrupted during mating. Protein profiles of the MAGs of *S. litura* revealed the occurrence of a protein below 14 kDa in virgin males and absence in interrupted mating males signifying the probability of the protein to be transferred to the female moths just before the end of mating [32]. Subsequently, using 2D gel electrophoresis, several proteins that were present in virgin males and are reduced or depleted completely in mated males were detected. The proteins predicted to be transferred to females during mating were further sequenced and categorized into six diverse functional groups such as metabolism, protein folding, antioxidation, olfaction, DNA binding/replication and cellular activity/response [33]. The latter data are useful as several mating-responsive genes and potential modulators of female behaviours were identified and characterized in *S. litura*.

These resources combined with our present study will be beneficial for future research on mating-responsive genes in *S. litura*. Future studies with the individual MAG protein or in combination will be required to understand the precise mechanisms by which these proteins alter female behaviour and reproduction. Thus, this study provides essential data to address fundamental questions about reproduction within and among insects and also paves the way for further exploration of the functions of these proteins in female insects. Finally, this new information on mating-responsive genes may be useful in the development of new insecticides to combat these pests that show high levels of resistance against the current classes of insecticides.

## Conclusions

Conclusively, a high-quality transcriptome was generated from the MAGs of *S. litura* by next-generation sequencing technology. Overall, 91,744 unigenes were generated, of which 53.09% of transcripts were annotated based on the sequence homology against the NR database. The GO function categories of these unigenes were associated with cellular components, biological processes and molecular functions. In total, 16,969 unigenes were differentially expressed between the two groups, among which 9814 were up-regulated and 7155 down-regulated. Several prominent genes including odorant binding protein, cytochrome P450, heat shock proteins, juvenile hormone binding protein, carboxypeptidases and serine protease were differentially expressed. Identification of all the key genes in this study may play different roles in post-mated females to ensure successful fertilization. Thus, our study provides essential data to address fundamental questions about reproduction within and among insects and also paves way for further exploration of the functions of these proteins in female insects.

## Data Availability

Not applicable.

## Abbreviations

MAG: Male accessory gland
NR: Non-redundant
RNA: Ribonucleic acid
NBAIR: National Bureau of Agricultural Insect Resources
NCBI: National Centre for Biotechnology Information
SDS-PAGE: Sodium dodecyl sulphate-polyacrylamide gel electrophoresis
2D: 2 dimensional
RIN: RNA integrity number
KAAS: KEGG Automatic Annotation Server
FPKM: Fragments per kb per million fragments
DEG: Differentially expressed genes
cDNA: Complementary deoxyribonucleic acid
GO: Gene ontology
OBP: Odorant binding protein
CYP: Cytochrome P450
Hsps: Heat shock proteins
JH: Juvenile hormone
JHBP: Juvenile hormone binding protein
ACE: Angiotensin I-converting enzyme
